# Botulinum toxin A for the Treatment of Overactive Bladder

**DOI:** 10.3390/toxins8030059

**Published:** 2016-02-29

**Authors:** Po-Fan Hsieh, Hung-Chieh Chiu, Kuan-Chieh Chen, Chao-Hsiang Chang, Eric Chieh-Lung Chou

**Affiliations:** 1 Department of Urology, China Medical University Hospital, Taichung 40447, Taiwan; phdoublem@yahoo.com.tw (P.-F.H.); b101091082@tmu.edu.tw (H.-C.C.); urology8395@yahoo.com.tw (C.-H.C.); 2 Department of Surgery, China Medical University Hospital, Taichung 40447, Taiwan; kuanchieh_c@hotmail.com; 3 School of Medicine, China Medical University, Taichung 40402, Taiwan

**Keywords:** botulinum toxin A, overactive bladder, detrusor overactivity

## Abstract

The standard treatment for overactive bladder starts with patient education and behavior therapies, followed by antimuscarinic agents. For patients with urgency urinary incontinence refractory to antimuscarinic therapy, currently both American Urological Association (AUA) and European Association of Urology (EAU) guidelines suggested that intravesical injection of botulinum toxin A should be offered. The mechanism of botulinum toxin A includes inhibition of vesicular release of neurotransmitters and the axonal expression of capsaicin and purinergic receptors in the suburothelium, as well as attenuation of central sensitization. Multiple randomized, placebo-controlled trials demonstrated that botulinum toxin A to be an effective treatment for patients with refractory idiopathic or neurogenic detrusor overactivity. The urinary incontinence episodes, maximum cystometric capacity, and maximum detrusor pressure were improved greater by botulinum toxin A compared to placebo. The adverse effects of botulinum toxin A, such as urinary retention and urinary tract infection, were primarily localized to the lower urinary tract. Therefore, botulinum toxin A offers an effective treatment option for patients with refractory overactive bladder.

## 1. Introduction

Overactive bladder (OAB) is a clinical diagnosis defined by the International Continence Society as “urgency, with or without urge incontinence, usually with frequency and nocturia” [1]. The overall prevalence of OAB was 12%–17% in the general population and it poses a great impact on daily activities and quality of life (QOL) [2,3,4,5]. In contrast to the clinical diagnosis of OAB, detrusor overactivity (DO) is a urodynamic observation characterized by involuntary detrusor contraction during the filling phase which may be spontaneous or provoked [1]. There is a substantial overlapping between OAB and DO, and DO is generally regarded as a major underlying physiology of OAB [6]. The standard treatment for OAB starts with patient education and behavior therapies. Among pharmacologic treatments, antimuscarinic agents are widely used but associated with adverse effects such as dry mouth, constipation, and blurred vision [7,8]. Due to the bothering side effects, the long-term adherence was consequently very low for antimuscarinic agents (12.0% to 39.4% at 12 months and 0% to 16% at 36 months) [9]. Beta3-adrenoceptor agonist is an alternative to antimuscarinic agents. The efficacy was similar among beta3-adrenoceptor agonists and antimuscaniric agents, but beta3-adrenoceptor agonists produce less rates of dry mouth and constipation [7].

Botulinum toxin was first isolated from *Clostridium botulinum* by van Ermengem [10]. There were seven serotypes of botulinum toxin, and botulinum toxin A (BoNT/A) was used most frequently in the urologic field. The efficacy of BoNT/A in treating OAB has been supported by literatures [11,12,13]. Currently both AUA and EAU guidelines suggested that intravesical injection of BoNT/A should be offered to patients with urgency urinary incontinence (UUI) refractory to antimuscarinic and beta3-adrenoceptor agonist therapy, and the FDA approved dose was 100 U of BoNT/A for idiopathic detrusor overactivity (IDO) and 200 U for neurogenic detrusor overactivity (NDO) [7,14]. In this article we will review the mechanism of BoNT/A in the treatment of OAB and evaluate randomized, placebo-controlled trials for the applicability of BoNT/A in IDO and NDO.

## 2. Mechanism of Action

BoNT/A is composed of a 100 kDa heavy chain polypeptide and a 50 kDa light chain polypeptide, as is joined via a disulfide bund [15]. The initially-proposed mechanism of BoNT/A was that by attachment of the heavy chain to the protein receptor SV2 on axon terminals, the toxin could enter the neuron by endocytosis [16]. Then the light chain cleaves synaptosomal-associated protein (25 kDa) (SNAP-25), a protein from the soluble *N*-ethylmaleimide-sensitive factor attachment receptor (SNARE) family. As a result, the fusion of neurosecretory vesicles and release of acetylcholine(Ach) from presynaptic nerve terminals are blocked [17]. With inhibition of Ach release, the effect on suburothelial afferent and detrusor parasympathetic nerve endings was abolished, which was similar to the action of anticholinergic agents [8,18]. However, emerging studies indicated that BoNT/A also exerts complex inhibitory effects on other neurotransmitters, neuropeptides, and receptors mediating neurotransmission [19]. In a rat model, BoNT/A injection was shown to reduce capsaicin or ATP-induced contraction frequencies and amplitudes [20]. Patients with DO had increased expression of the capsaicin receptor TRPV1 and purinergic receptor P2X3 in the suburothelial nerve fibers, and intradetrusor injection of BoNT/A resulted in decreased levels of these sensory receptors [21,22,23]. The change in P2X3 immunoreactivity was correlated with improved sensation of urgency in patients with intractable neurogenic or idiopathic DO [23]. Besides, women with DO had increased densities of suburothelial substance P (SP) and calcitonin gene related peptide (CGRP) immunoreactive fibers [24]. BoNT/A injection could reduce the release of CGRP with improvement in the intervals between bladder contractions in a rat model [25]. Inhibition of SP by BoNT/A would reduce the activation of TRPV1 and P2X3 receptors in suburothelium and detrusor muscle via decreased activation of NK1 receptors as was shown in a guinea pig model [26]. In addition, intravesical BoNT/A leaded to neurotrophic growth factors (NGF) deprivation up to 3 months, which was correlated with clinical response in patients with DO [27,28]. Last but not least, a recent study showed that there was a significant accumulation of the radiolabelled BoNT/A in the lumbosacral dorsal root ganglia following intradetrusor injection in rats [29]. In other words, BoNT/A works through not only localized peripheral mechanism but also a CNS effect through retrograde axonal transport.

In summary, it was proposed that the action of BoNT/A on human overactive bladders followed a sequential mechanism [19]. First, the immediate effect of BoNT/A is inhibition of vesicular release of Ach, ATP, and SP and the axonal expression of TRPV1 and P2X3, leading to peripheral denervation [18,30,31]. Second, after SNAP-25 was cleaved by BoNT/A, this exocytosis inhibitor could persist for a long time (up to 40 days in a mouse model) [17]. Third, through retrograde transport into CNS BoNT/A would result in attenuation of central sensitization, leading to further peripheral desensitization.

## 3. Application of Botulinum Toxin A

### 3.1. Idiopathic Detruosr Overacitity

Radziszewski *et al*. conducted the first trial of BoNT/A for patients with IDO. All seven patients became continent after treatment with 300 U of BoNT/A [32]. In November 2015, we searched randomized, placebo-controlled trials of BoNT/A on Pubmed using the keywords “Botulinum toxin A” and “detrusor overactivity”. Relevant literature in the reference lists were also reviewed. Finally, eleven randomized, placebo-controlled trials of BoNT/A for IDO to date were identified and summarized in Table 1 [33,34,35,36,37,38,39,40,41,42,43]. The definition of refractory IDO/OAB varied among studies. Most studies enrolled patients with OAB symptoms inadequately responded to anticholinergic therapy or those who had intolerable side effects [33,35,37,38,39,40,41,42,43]. Failed behavioral modification in addition to anticholinergic therapy was used as inclusion criteria in two studies [34,36].

Sahai *et al.* reported the first randomized, double-blind, placebo-controlled trial to compare the efficacy of BoNT/A *versus* placebo in treating patients with refractory IDO of either sex [33]. BoNT/A of 200 U (10 U/mL) was injected at 20 sites with trigone sparing. Significant increases in maximum cystometric capacity (MCC) from 182 mL to 313 mL were observed at 4 weeks. BoNT/A also reduced episodes of frequency (mean change from 15.44 to 7.93 times per day), urgency (mean change from 11.69 to 9.21 times per day) as well as UUI (mean change from 4.98 to 1.9 times per day) at 4 weeks, and a significantly better improvement in QOL as compared with placebo was noted. The beneficial effects persisted for at least 24 weeks. Brubaker *et al.* also compared 200 U intradetrusor BoNT/A injection at 15 to 20 sites *versus* placebo in women with refractory idiopathic UUI [34]. Based on the Patient Global Impression of Improvement, they found a greater clinical response rate and a greater reduction in mean incontinence episodes in those who received BoNT/A compared to those who received placebo. The median duration of responses was 373 days, significantly longer than 62 days for placebo. Later Tincello *et al.* conducted a larger study to evaluate the efficacy of BoNT/A 200 U for the treatment of refractory IDO [35]. In this study, 122 women received BoNT/A injection at 20 sites and 118 women received placebo. In the treatment group, patients experienced greater reduction of median voiding frequency, urgency episodes, and leakage episodes compared with placebo (difference 1.34, 2.50, and 4.33 respectively). Continence was more common after BoNT/A injection. Flynn *et al.* used 200 U and 300 U BoNT/A of higher concentration (66–100 U/mL in 10–12 sites) *versus* placebo to treat 22 female patients with refractory OAB [36]. The combined results of the two different doses showed that there were significant improvements in daily incontinence episodes (mean change from 7.9 to 3.4 times), pads changed per day (mean change from 4.4 to 2.2) and QOL questionnaires in the treatment group.

On the other hand, some trials were designed to evaluate the benefit of BoNT/A 100 U in the treatment of refractory IDO. In a small-scaled study (10 BoNT/A *versus* 11 placebo), Dowson *et al.* reported that BoNT/A could significantly increase the mean MCC by 105 mL, but the storage symptoms and QOL remained statistically unchanged following BoNT/A 100 U injection [37]. However, Chapple *et al.* conducted a study of BoNT/A 100 U *versus* placebo in 558 patients of either sex and showed different results [43]. BoNT/A 100 U not only decreased urinary incontinence (UI) episodes (mean change from 5.5 to 2.55 times per day for BoNT/A *versus* mean change from 5.7 to 4.67 times per day for placebo) at 12 weeks but also improved the treatment benefit scale, other OAB symptoms (episodes of UUI, micturition, urgency, and nocturia) and health-related quality of life (HRQOL). Nitti *et al.* also tested BoNT/A 100 U *versus* placebo in 557 patients of either sex with idiopathic OAB and confirmed the benefit of BoNT/A in decreasing daily frequency of UI episodes vs placebo (mean change from 5.5 to 2.85 times per day for BoNT/A *versus* mean change from 5.1 to 4.23 times per day for placebo) [38]. All other OAB symptoms including frequency, urgency, and nocturia episodes as well as HRQOL were also improved greater by BoNT/A 100 U compared with placebo.

To assess the dose response relationship of intradetrusor BoNT/A, Dmochowski *et al.* conducted a trial using BoNT/A of doses ranging from 50 U to 300 U administered as 20 intradetrusor injections for male and female patients with idiopathic OAB and UUI [39]. They found durable efficacy for all BoNT/A dose groups of 100 U or greater. However, doses greater than 150 U contributed minimal additional or clinically relevant improvement in symptoms and HRQOL. On the other hand, use of clean intermittent catheterization (CIC) was dose dependent. Therefore, 100 U of BoNT/A may be the appropriate dose balancing the benefits and safety profiles. Rovner *et al.* tested the same dose range and also found that dose of 100 U or more led to significant improvements in OAB symptoms [40]. Dose-response relationship was observed in the changes of mean MCC and the volume at first involuntary detrusor contraction. Fowler *et al.* confirmed that BoNT/A at doses of 100 U or more produced significantly greater improvements than placebo in HRQOL by week 2, and the effect could sustain for up to 36 weeks [41]. Denys *et al.* performed a trial to evaluate the efficacy and tolerability of low doses of BoNT/A (50 U, 100 U and 150 U) compared to placebo in patients with idiopathic OAB [42]. They reported more than 50% improvement *versus* baseline in urgency and UUI at month 3 in 65% and 56% patients receiving 100 U and 150 U BoNT/A injections. Complete continence was achieved in 55% and 50% patients after 100 U and 150 U BoNT/A, respectively. These benefits persisted up to 6 months.

Recently, a meta-analysis including eight publications to evaluate the safety and efficacy of BoNT/A in treating idiopathic OAB was published [12]. It was reported that BoNT/A significantly decreased the mean number of UI per day (−2.77 *versus* −1.01, the standard mean difference [*SMD*] = −1.68) and the mean number of micturitions per day (−1.61 *versus* −0.87, *SMD* = −1.82); increased MCC (91.39 *versus* 32.32, *SMD* = 63.82) and volume voided (44.29 *versus* 7.36, *SMD* = 33.05) compared with placebo. Besides, 29.20% of patient treated with BoNT/A became incontinence-free *versus* 7.95% of patients with placebo (odds ratio [*OR*] = 4.89). In addition, the injection method of all the above studies was intradetrusor injection with trigone sparing in order to reduce the potential complication of vesicoureteral reflux. However, two RCTs by Kuo *et al.* and Manecksha *et al.* revealed that bladder base/trigone injection of BoNT/A was as safe and effective as bladder body injections with or without trigone involvement [44,45].

### 3.2. Neurogenic Detruosr Overacitity

In 2000, Schurch *et al.* reported a preliminary study of BoNT/A in treating NDO patients. In their initial experience, BoNT/A of 200 U to 300 U significantly increased MCC and decreased detrusor voiding pressure, and complete continence was restored in 17 of 19 cases by 6 weeks [46]. On searching with “Botulinum toxin A” and “detrusor overactivity” on Pubmed in November 2015 and reviewing relevant articles in the reference lists, we identified that at present there are four randomized, placebo-controlled trials which examined the efficacy of BoNT/A for NDO (Table 2) [47,48,49,50].

Schurch *et al*. conducted the first randomized trial to determine the efficacy of two doses of BoNT/A (200 U and 300 U administered as 30 intradetrusor injections) in patients with urinary incontinence caused by NDO [47]. Both BoNT/A treatment groups had significantly decreased UI episodes (mean change −58% and −54% for 200 U and 300 U respectively), increased MCC (mean change 174.2 mL and 92.9 mL for 200 U and 300 U respectively), and improved QOL. There was no clinically relevant difference between the two doses of BoNT/A. The other two clinical trial by Cruz *et al.* and Ginsberg *et al.* also tested 200 U and 300 U BoNT/A in patients with NDO [48,49]. Significantly greater improvements in UI episodes, QOL, and urodynamic parameters including MCC and maximum detrusor pressure during the first involuntary detrusor contraction compared with placebo were noted in both doses of BoNT/A in both studies. Again, both 200 U and 300 U of BoNT/A yielded similar efficacy and duration of benefits.

Consistent with above findings, Herschorn *et al.* performed a trial using BoNT/A 300 U with intradetrusor injection at 30 sites for NDO patients [50]. After injection of BoNT/A, there was a lower frequency of UI episodes, greater improvements in urodynamic and QOL parameters compared with placebo. Interestingly, the clinical benefits lasted for up to 9 months.

Recently, a meta-analysis including the four RCTs was published [13]. It was revealed that BoNT/A resulted in changes of the mean number of UI per week (*SMD* = −10.91), MCC (*SMD* = 146.09), and maximum detrusor pressure (*SMD* = −32.65). Regardless of the doses of 200 U or 300 U, BoNT/A was more effective than placebo in treating patients with NDO.

## 4. Adverse Events

According the meta-analysis of BoNT/A in treating IDO patients, BoNT/A significantly increased PVR (32.77 *vs.* 2.01, *SMD* = 31.73), proportions of urinary tract infection (UTI) (19.69% and 5.94%, *OR* = 3.89), and proportions of CIC (8.41% and 0.46%, *OR* = 13.39) *versus* placebo [12]. In treating NDO patients, BoNT/A also led to a higher risk of UTI (relative risk [*RR*] = 1.48), hematuria (*RR* = 1.81), and urinary retention (*RR* = 5.87) requiring catheterization [13]. The effect of increased post-void residual volume (PVR) was dose dependent, and doses >150U was found to be more commonly associated with *PVR* > 200 mL [39,40]. It was reported that age >61 years, low maximum flow rate, low voiding efficiency (a percentage of the voided volume compared to the prevoid bladder volume <90%), and large PVR at baseline were risk factors for these adverse events [51]. The effect of increased PVR peaked by 2 weeks following BoNT/A injection, with a gradual decrease thereafter [39,49]. For urinary retention requiring catheterization, the duration was 6 weeks or less in more than half of the patients [12]. On the other hand, a meta-analysis showed there was no significant difference between trigonal and extratrigonal BoNT/A injection in terms of adverse events [52]. Kuo *et al.* demonstrated in a pilot study that intravesical instillation of liposome-encapsulated BoNT/A could effectively reduce the episodes of frequency and urgency in OAB patients without any increase in PVR or risk of UTI [53]. The promising role of liposome as vehicles delivering BoNT/A should be further validated. Finally, these results indicated that the adverse events of BoNT/A were primarily localized to the urinary tract, in contrast to the systemic effects of anticholinergic agents. Although muscle weakness or hyposthenia has been reported after BoNT/A injection, the incidence of systemic adverse events was rare and the duration was transient [54,55].

## 5. Conclusions

By blockade of neurotransmitter release and suburothelial sensory receptors expression, BoNT/A could cause chemical denervation of detrusor muscle. For patients with refractory OAB, BoNT/A offers an effective treatment option and the adverse events were primarily localized to the lower urinary tract.

## Figures and Tables

**Table 1:** Randomized controlled trials of botulinum toxin A for idiopathic detrusor overactivity.

Authors/Year	No. Patients	Injection Method	UI Episode (%Change)	Frequency (%Change)	MCC (%Change)	Pdetmax (%Change)	Urinary Retention Requiring CIC (%)	Duration of Benefit
Sahai *et al.*/2006 [33]		Intravesical injection (trigone sparing)						
Placebo	18		−18	−7	−15	−4	None	NA
Botox 200 U	16		−70	−49	+72	−59	37.5%	>24 weeks
Brubaker *et al.*/2008 [34]		Intravesical injection (trigone sparing)						
Placebo	15		−5	NA	NA	NA	None	62 days
Botox 200 U	28		−75	NA	NA	NA	43%	373 days
Flynn *et al.*/2009 [36]		Intravesical injection (trigone sparing)						
Placebo	7		9.3	−6.8	NA	NA	None	NA
Botox 200 U or 300 U	15		−57.5	−12.2	NA	NA	7	>6 weeks
Dmochowski *et al.*/2010 [39]		Intravesical injection (trigone sparing)						
Placebo	44		−17.4	NA	NA	NA	0	NA
Botox 50 U	56		−20.7	NA	NA	NA	5.4	18 weeks
Botox 100 U	55		−18.4	NA	NA	NA	10.9	24 weeks
Botox 150 U	50		−23	NA	NA	NA	20	36 weeks
Botox 200 U	52		−19.6	NA	NA	NA	21.2	36 weeks
Botox 300 U	55		−19.4	NA	NA	NA	16.4	36 weeks
Dowson *et al.*/2010 [37]		Intravesical injection (trigone sparing)						
Placebo	13		45	0	0	NA	0	12 weeks
Botox 100 U	10		−8.3	−4.5	+40.9	NA	30	12 weeks
Rovner *et al.*/2011 [40]		Intravesical injection (trigone sparing)						
Placebo	44		−51.7	−15.6	+4.7	−10.7	0	NA
Botox 50 U	57		−69.9	−23.9	+8	0	3.6	12 weeks
Botox 100 U	54		−64.6	−26.4	+15.1	+24	9.1	12 weeks
Botox 150 U	49		−89.6	−14.4	+19.3	+3.4	12.7	36 weeks
Botox 200 U	53		−80	−24.8	+17.2	+5.5	18.2	36 weeks
Botox 300 U	56		−77.6	−27.1	+21.8	+23.1	16.4	36 weeks
Fowler *et al.*/2012 [41]		Intravesical injection (trigone sparing)						
Placebo	44		−53.5	NA	NA	NA	0	NA
Botox 50 U	57		−68.3	NA	NA	NA	14.6	12 weeks
Botox 100 U	54		−66.2	NA	NA	NA	-	24–36 weeks
Botox 150 U	49		−81.3	NA	NA	NA	-	24–36 weeks
Botox 200 U	53		−73	NA	NA	NA	-	24–36 weeks
Botox 300 U	56		−72.3	NA	NA	NA	-	24–36 weeks
Denys *et al.*/2012 [42]		Intravesical injection (trigone sparing)						
Placebo	31		29% had UUI reduction > 50%		+10.5	−49.3	3.2	NA
Botox 50 U	23		37% had UUI reduction > 50%	NA	+8	−51.2	13	6 months
Botox 100 U	23		65% had UUI reduction > 50%	NA	+8	−36.5	4.3	6 months
Botox 150 U	30		56% had UUI reduction > 50%	NA	+9.5	-49.5	13.3	6 months
Tincello *et al.*/2012 [35]		Intravesical injection (trigone sparing)						
Placebo	118		−3.2	−9.6	NA	NA	4	NA
Botox 200 U	122		−73.1	−19.1	NA	NA	16	6 months
Chapple *et al.*/2013 [43]		Intravesical injection (trigone sparing)						
Placebo	271		−13.9	-6	NA	NA	0.7	NA
Botox 100 U	277		−53.2	−19.7	NA	NA	6.9	12 weeks
Nitti *et al.*/2013 [38]		Intravesical injection (trigone sparing)						
Placebo	277		-17.1	4.1	NA	NA	0.4	NA
Botox 100 U	280		-48.2	−16.9	NA	NA	5.4	12 weeks

**Table 2:** Randomized controlled trials of botulinum toxin A for neurogenic detrusor overactivity.

Authors/Year	No. of Patients	Patients Type	Injection Method	Continence (%)/ UI Episode (%Change)	MCC (%Change)	Pdetmax (%Change)	Urinary Retention Requiring CIC (%)	Duration of Benefit
Schurch *et al.*/2005 [46]		53 SCI, 6 MS	Intravesical injection (trigone sparing)					
Placebo	21			24/−10	+18	−13	-	NA
Botox 200 U	19			71/−58	+85	−59	-	>24 weeks
Botox 300 U	19			53/−54	+63	−56	-	>24 weeks
Cruz *et al.*/2011 [47]		121 SCI, 154 MS	Intravesical injection (trigone sparing)					
Placebo	92			7.6/−36	+3	+15	12	13.1 week
Botox 200 U	92			38.0/−67	+64	−55	30	42.1 week
Botox 300 U	91			39.6/−62	+64	−64	42	42.1 week
Herschorn *et al.*/2011 [49]		38 SCI, 19 MS	Intravesical injection (trigone sparing)					
Placebo	29			NA/0	−21.9	+4	-	NA
Botox 300 U	29			NA/−25	+21.5	+49.1	-	9 months
Ginsberg *et al.*/2012 [48]		189 SCI, 227 MS	Intravesical injection (trigone sparing)					
Placebo	149			NA/−31.1	+6.3	−4.7	10	92 days
Botox 200 U	135			NA/−65	+59.9	−68.4	35	256 days
Botox 300 U	132			NA/−73	+65.6	−70.7	42	254 days

## Abbreviations

The following abbreviations are used in this manuscript:

OAB	overactive bladder
QOL	quality of life
DO	detrusor overactivity
BONT/A	botulinum toxin A
UUI	urgeny urinary incontinence
IDO	idiopathic detrusor overactivity
NDO	neurogenic detrusor overactivity
Ach	acetylcholine
MCC	maximum cystometric capacity
UI	urinary incontinence
HRQOL	health-related quality of life
CIC	clean intermittent catheterization
PVR	post-void residual volume
UTI	urinary tract infection
